# Comparison Between Fine Needle Aspiration and Core Needle Biopsy for the Diagnosis of Thyroid Nodules: Effective Indications According to US Findings

**DOI:** 10.1038/s41598-020-60872-z

**Published:** 2020-03-18

**Authors:** Soo Yeon Hahn, Jung Hee Shin, Young Lyun Oh, Ko Woon Park, Yaeji Lim

**Affiliations:** 1 Department of Radiology and Center for Imaging Science, Samsung Medical Center, Sungkyunkwan University School of Medicine, 81 Irwon-ro, Gangnam-gu, Seoul 06351 Republic of Korea; 2 Department of Pathology, Samsung Medical Center, Sungkyunkwan University School of Medicine, 81 Irwon-ro, Gangnam-gu, Seoul 06351 Republic of Korea; 3 Department of Applied Statistics, Chung-Ang University, 84, Heukseok-ro, Dongjak-gu, Seoul 06974 Republic of Korea

**Keywords:** Thyroid cancer, Cancer imaging

## Abstract

Thyroid nodules are initially handled by fine needle aspiration (FNA). However, the stance of thyroid core needle biopsy (CNB) still is a challenge. This study aimed to compare the diagnostic performances and conclusive rates of FNA and CNB for the diagnosis of thyroid nodules and to define effective indications of CNB. This retrospective study enrolled 1,060 consecutive thyroid nodules in 1,037 patients who underwent FNA from January 2008 to May 2008, and 462 consecutive nodules in 453 patients who underwent CNB from January 2014 to December 2015 at our institution. Ultrasound (US) features were classified according to the American College of Radiology Thyroid Imaging Reporting and Data System (ACR TI-RADS) and Korean TIRADS (K-TIRADS). We compared diagnostic performances and conclusive rates between FNA and CNB groups. Propensity score matching was conducted to match FNA patients with CNB patients. After matching, the diagnostic performances for selecting surgical candidates and predicting malignancy were comparable between the two biopsy groups. Based on US findings, conclusive results were obtained significantly more in CNB than in FNA when thyroid nodules were classified as ACR TI-RADS or K-TIRADS category 4 and measured larger than 2 cm. Diagnostic performances between FNA and CNB were comparable. Superiority of CNB to FNA was found for thyroid nodules larger than 2 cm and classified as ACR TI-RADS or K-TIRADS category 4.

## Introduction

Fine needle aspiration (FNA) under ultrasound (US) guidance for thyroid nodules is considered the standard diagnostic tool due to its advantages including simplicity, safety, cost-effectiveness, and high diagnostic specificity. Several FNA guidelines for thyroid nodules have been published by various research groups over the years. Although there are slight differences in their recommendations, the indications for US-guided FNA in thyroid nodules have been well established by these authoritative guidelines^1–7^.

Core needle biopsy (CNB) for thyroid nodules has been suggested as an additional diagnostic method to US-guided FNA, to overcome the diagnostic limitations including non-diagnostic or inconclusive results and to prevent repeat FNA or unnecessary surgery^8–13^. Han *et al*. suggested the modified core biopsy technique to increase diagnostic yields for well-circumscribed indeterminate thyroid nodules^14^. In addition, our previous study proposed the ideal core number for US-guided CNB of cytologically inconclusive thyroid nodules^11^. In 2015, the Korean Endocrine Pathology Thyroid Core Needle Biopsy Study Group developed a microscopic reporting system for thyroid CNB based on the six-level reporting categories of the Bethesda System for Reporting Thyroid Cytopathology^15,16^. In 2016, the Korean Society of Thyroid Radiology revised the consensus statement and recommendations for CNB of the thyroid nodules^17^.

In addition, previously a few studies with propensity score methods performed to compare of CNB and FNA. One study demonstrated that CNB was more accurate and sensitive and had less false negative and non-diagnostic results^18^. Meanwhile, another study showed that CNB had a higher specificity for predicting neoplasm compared with FNA^19^. However, until now, the obvious indications of CNB have not yet been clearly established.

Therefore, the purpose of this study was to compare the diagnostic performances and conclusive rates of FNA and CNB for the diagnosis of thyroid nodules and to define effective indications of CNB.

## Results

### Clinicopathological and US characteristics

Table 1 shows the clinicopathological and US characteristics of FNA and CNB patients before and after matching. For both groups, only age was comparable before matching (*P* = 0.149). In view of Bethesda or CNB diagnostic categories, none of the patients demonstrated non-diagnostic CNB results, while 13.9% of patients showed non-diagnostic FNA results. The incidences of the atypia/follicular lesion of undetermined significance (AUS/FLUS) or indeterminate, follicular neoplasm/suspicious for follicular neoplasm (FN/SFN), and suspicious for malignancy categories were higher in the CNB group than in the FNA group. The proportions of the benign and malignant categories were higher in the FNA group than in the CNB group (*P* < 0.001). CNB Patients had more nodules that were larger than 2 cm (*P* < 0.001), with solid composition (*P* < 0.001), ACR TI-RADS categories 3 and 4 (*P* < 0.001), and K-TIRADS category 4 (*P* < 0.001) compared with FNA patients.

**Table 1:** Clinicopathological and ultrasonographic characteristics of patients with FNA or CNB before and after matching.

	Before matching			After matching		
	FNA (*n* = 1060)	CNB (*n* = 462)	*P* Value	FNA (*n* = 171)	CNB (*n* = 171)	*P* Value
Mean age ± SD	51.01 ± 12.46	49.98 ± 13.20	0.149	49.88 ± 12.34	50.39 ± 13.39	0.718
Sex			0.014*			0.815
Female	823 (79.4)	333 (73.7)		117 (68.4)	120 (70.2)	
Male	213 (20.6)	119 (26.3)		54 (31.6)	51 (29.8)	
Present Bethesda or CNB diagnostic category			<0.001*			0.066
I (non-diagnostic)	147 (13.9)	0		6 (3.5)	0	
II (benign)	728 (68.7)	168 (36.4)		91 (53.2)	91 (53.2)	
III (AUS/FLUS)	50 (4.7)	41 (8.9)		23 (13.5)	21 (12.3)	
IV (FN/SFN)	4 (0.4)	193 (41.8)		3 (1.8)	8 (4.7)	
V (suspicious for malignancy)	14 (1.3)	10 (2.2)		2 (1.2)	7 (4.1)	
VI (malignant)	117 (11.0)	50 (10.8)		46 (26.9)	44 (25.7)	
Conclusive diagnosis	863 (81.4)	421 (91.1)	<0.001*	142 (83.0)	150 (87.7)	0.302
Previous Bethesda or CNB diagnostic category			<0.001*			<0.001*
Not done	826 (77.9)	170 (36.8)		119 (69.6)	75 (43.9)	
I	63 (5.9)	37 (8.0)		10 (5.8)	10 (5.8)	
II	135 (12.7)	95 (20.6)		22 (12.9)	34 (19.9)	
III	34 (3.2)	141 (30.5)		18 (10.5)	40 (23.4)	
IV	1 (0.1)	5 (1.1)		1 (0.6)	1 (0.6)	
V	0	11 (2.4)		0	8 (4.7)	
VI	1 (0.1)	3 (0.6)		1 (0.6)	3 (1.8)	
Mean nodule size ± SD	2.12 ± 1.12	2.65 ± 1.35	<0.001*	2.39 ± 1.16	2.65 ± 1.43	0.077
Range of nodule size			<0.001*			0.820
≤2 cm	603 (56.9)	183 (39.6)		71 (41.5)	74 (43.3)	
>2 cm	457 (43.1)	279 (60.4)		100 (58.5)	97 (56.7)	
Nodule composition on US			<0.001*			0.500
Predominantly cystic	71 (6.7)	4 (0.9)		2 (1.2)	4 (2.3)	
Predominantly solid	424 (40.0)	117 (25.3)		52 (30.4)	45 (26.3)	
Solid	565 (53.3)	341 (73.8)		117 (68.4)	122 (71.3)	
ACR TI-RADS category			<0.001*			0.712
2	378 (35.7)	93 (20.1)		36 (21.1)	41 (24.0)	
3	334 (31.5)	158 (34.2)		50 (29.2)	41 (24.0)	
4	220 (20.8)	170 (36.8)		49 (28.7)	53 (31.0)	
5	128 (12.1)	41 (8.9)		36 (21.1)	36 (21.1)	
K-TIRADS category			<0.001*			1.000
3	771 (72.7)	290 (62.8)		93 (54.4)	90 (52.6)	
4	179 (16.9)	133 (28.8)		45 (26.3)	48 (28.1)	
5	110 (10.4)	39 (8.4)		33 (19.3)	33 (19.3)	

Note:— Data in parentheses are percentages.

SD, standard deviation; AUS/FLUS, atypia of undetermined significance or follicular lesion of undetermined significance; FN/SFN, follicular neoplasm or suspicious for follicular neoplasm; ACR TI-RADS, American College of Radiology Thyroid Imaging Reporting and Data System; K-TIRADS, Korean Thyroid Imaging Reporting and Data System.

*A *P* Value of <0.05 was considered statistically significant.

After FNA patients matching with CNB patients, regarding the matched variables, no differences were found between the two groups, except for previous FNA or CNB results (*P* < 0.001).

### Diagnostic performances of FNA and CNB

Table 2 presents diagnostic performances of FNA and CNB to predict malignancy and to determine surgical indication (i.e. Bethesda or CNB diagnostic category IV, V, and VI).

**Table 2:** Diagnostic performances of FNA and CNB to predict malignancy and to determine surgical indication.

	Before matching			After matching		
	FNA	CNB	*P* Value	FNA	CNB	*P* Value
**For malignancy prediction**						
Sensitivity	89.6 (82.5–94.5)	93.4 (84.1–98.2)	0.583	98.3 (88.9–99.8)	92.9 (39.9–99.6)	0.187
Specificity	98.7 (96.9–99.6)	100 (95.9–100)	0.589	49.2 (48.2–50.1)	50.0 (48.7–51.3)	0.083
PPV	95.4 (89.5–98.5)	100 (93.7–100)	0.165	95.3 (87.1–98.4)	100	> 0.999
NPV	96.9 (94.6–98.4)	95.7 (89.2–98.8)	0.526	98.9 (92.5–99.8)	95.6 (52.9–99.8)	0.212
Accuracy	96.5 (94.5–98.0)	97.3 (93.3–99.3)	0.796	78.8 (75.3–79.8)	74.8 (57.8–77.5)	0.178
**For surgical indication**						
Sensitivity	77.8 (69.8–84.5)	89.7 (82.8–94.6)	0.011*	93.8 (84.5–97.6)	83.6 (51.4–96.1)	0.078
Specificity	98.8 (97.3–99.5)	54.9 (48.0–61.7)	<0.001*	96.6 (91.3–98.7)	96.5 (83.1–99.4)	0.970
PPV	94.6 (88.6–98.0)	52.2 (45.1–59.3)	<0.001*	93.7 (84.5–97.6)	93.3 (70.8–98.8)	0.925
NPV	94.1 (91.7–96.0)	90.7 (84.3–95.1)	0.166	96.6 (91.3–98.7)	91.0 (68.4–97.9)	0.083
Accuracy	94.2 (92.1–95.9)	67.3 (61.9–72.3)	<0.001*	79.9 (76.3–81.7)	76.5 (64.0–81.8)	0.078

Note:— Values are percentages, and data in parentheses are 95% confidence intervals.

PPV, positive predictive value; NPV, negative predictive value.

*A *P* Value of <0.05 was considered statistically significant.

Before matching, FNA had a significantly higher specificity, positive predictive value (PPV), accuracy (*P*s < 0.001) and a lower sensitivity (*P* = 0.011) than CNB for selecting surgical candidates. Before matching, no differences were found in diagnostic performances between the two biopsy groups for predicting malignancy.

After matching, the diagnostic performances for selecting surgical candidates and predicting malignancy were comparable between the FNA and CNB groups.

### Conclusive rates of FNA and CNB

Conclusive rates of FNA and CNB (Bethesda or CNB diagnostic categories II, IV, V, and VI vs. I and III) were also investigated and compared according to the final US category and nodule size. Depending on the degree of suspicion, conclusive results were obtained significantly more with CNB than with FNA when thyroid nodules were classified as ACR TI-RADS or K-TIRADS category 4 both before and after matching. For the subgroup analysis based on nodule size, the same results were also obtained for thyroid nodules larger than 2 cm after matching (Table 3).

**Table 3:** Conclusive results of FNA and CNB depending on the degree of sonographic suspicion and nodule size.

	Before matching			After matching								
				All sized nodules			Nodules ≤ 2.0 cm in size			Nodules > 2.0 cm in size		
	FNA	CNB	*P* Value	FNA	CNB	*P* Value	FNA	CNB	*P* Value	FNA	CNB	*P* Value
**K-TIRADS**												
3	638 (82.7)	253 (87.2)	0.092	75 (80.6)	73 (81.1)	0.936	22 (84.6)	19 (79.2)	0.619	53 (79.1)	54 (81.8)	0.693
4	125 (69.8)	129 (97.0)	<0.001*	35 (77.8)	44 (91.7)	0.071	19 (86.4)	23 (85.2)	0.308	16 (69.6)	21 (100)	0.009*
5	100 (90.9)	39 (100)	0.115	32 (97.0)	33 (100)	0.317	22 (92.3)	23 (100)	0.317	10 (100)	10 (100)	>0.999
**ACR TI-RADS**												
2	316 (83.6)	84 (90.3)	0.144	33 (91.7)	33 (80.5)	0.173	10 (83.3)	2 (100)	0.158	23 (95.8)	31 (79.5)	0.104
3	273 (81.7)	135 (85.4)	0.373	37 (74.0)	34 (82.9)	0.309	13 (94.9)	15 (83.3)	0.401	24 (66.7)	19 (82.6)	0.158
4	165 (75.0)	162 (95.3)	<0.001*	37 (75.5)	48 (90.6)	0.048*	17 (81.0)	24 (82.8)	0.871	20 (71.4)	24 (100)	0.005*
5	109 (85.2)	40 (97.6)	0.063	35 (97.2)	35 (97.2)	>0.999	23 (95.8)	22 (96.0)	0.976	12 (100)	11 (100)	>0.999
Total	863 (81.4)	421 (91.1)	<0.001*	142 (83.0)	150 (97.7)	0.223						

Note:— Data in parentheses are percentages.

ACR TI-RADS, American College of Radiology Thyroid Imaging Reporting and Data System; K-TIRADS, Korean Thyroid Imaging Reporting and Data System.

*A *P* Value of <0.05 was considered statistically significant.

## Discussion

We compared the diagnostic performances and conclusive rates of FNA and CNB performed at our institution for patients who had thyroid nodules equal to or larger than 1 cm. For diagnostic performance, FNA had a significantly higher specificity, PPV, and accuracy and a lower sensitivity than CNB for selecting surgical candidates before matching. In the other conditions, however, no significant differences were found in the diagnostic performances of the two biopsy groups. Meanwhile, the conclusive rates were significantly higher in CNB than in FNA when thyroid nodules were classified as ACR TI-RADS or K-TIRADS category 4 and were larger than 2 cm.

Previous literatures demonstrated significantly lower non-diagnostic result rates or higher accuracies for malignancy in CNB groups compared to FNA groups when the nodules showed previous non-diagnostic or previous indeterminate FNA results^8,10,13,20^. In addition, several studies have demonstrated that CNB was more effective even for initially detected thyroid nodules on US^18,21,22^. In this study, consistent with those studies, there was no non-diagnostic case of CNB, while non-diagnostic rates for FNA were 13.9% and 3.5% before and after matching, respectively. In addition, the diagnostic performances for malignancy were comparable between the two biopsy methods.

Conversely, other studies have reported that CNB has comparable or lower accuracy and sensitivity compared to FNA^17,23^. Among these studies, a recent study by Kim *et al*. comparing the diagnostic performances of 3,048 FNA and 144 CNB cases concluded that CNB may not be helpful in diagnosing papillary carcinomas and neoplasms^17^. However, despite their large sample size, only 16.3% of all patients underwent the procedures at the authors’ institution, while the remaining 2,672 patients (87.4% in total; 83.3% of FNA and 93.1% of CNB) were diagnosed by reviewing submitted slides after procedures at other hospitals. As is well known, the technique of FNA and CNB is one of the most important factors affecting the diagnostic yield of biopsy. In this study, we included only FNA or CNB cases performed at our institution in order to consistently compare the quality of the procedure. In addition, they did not include any nondiagnostic lesions by either FNA or CNB after matching for their comparative analysis of FNA and CNB groups. These two might be the significant limitations of their research.

Both CNB and FNA procedures under US guidance should be done by experienced operators who are familiar with the radiologic anatomy of the neck to avoid complications^24^. In particular, with CNB, the main concerns most commonly include bleeding and hematoma^24,25^. The CNB complication rates have been reported between 0.2% and 1.0%^25–28^, and patient discomfort and tolerability levels were not significantly different between FNA and CNB^28,29^. Fortunately, in our results, the superiority of CNB to FNA was apparent in nodules more than 2 cm with US findings categorized as intermediate suspicion. This indication reduces the risk of overuse of CNB in small thyroid nodules.

This study has several limitations. First, the retrospective data collection may have resulted in case selection bias. Second, there was a timing difference between the two biopsy groups, because CNB started to be actively performed in the middle of 2013 and the time when FNA had the least influence on CNB was 2008. Third, the proportion of ‘previous Bethesda or CNB diagnostic category III’ in CNB group was decreased after matching, which was still significantly higher than that in FNA group. As widely known, Bethesda or CNB diagnostic category III at initial biopsy is a risk factor leading repeated category III biopsy results. However, in this study, these did not affect the interpretation of our results. Forth, we did not compare the frequency and degree of complications with FNA and CNB groups. The frequency and degree of complications in FNA cases have been reported to be lower or similar compared with CNB cases. In our study, the complication rate of CNB was 0%. Last, in this study, we defined benign thyroid nodules as nodules with no significant changes in one-year follow-up and with benign FNA or CNB results. However, one year may not be enough to detect significant changes in certain nodules to distinguish benign nodules from malignant nodules.

In conclusion, our study revealed that CNB showed comparable diagnostic performance to FNA. Meanwhile, the conclusive rates were significantly higher in CNB than in FNA when thyroid nodules were classified as ACR TI-RADS or K-TIRADS category 4 and measured larger than 2 cm. Therefore, we can predict the effective indications of CNB for thyroid nodules that are larger than 2 cm and show US findings corresponding to ACR TI-RADS or K-TIRADS category 4.

## Materials and Methods

### Patient population

The Institutional Review Board at Samsung Medical Center, Seoul, Korea, approved this retrospective study, and patient approval and informed consent were not required for the review of US images and medical data. However, written informed consent was acquired from patients before undergoing the US-guided FNA and US-guided CNB procedures. In addition, all methods were conducted according to relevant guidelines and regulations.

**Figure 1:**
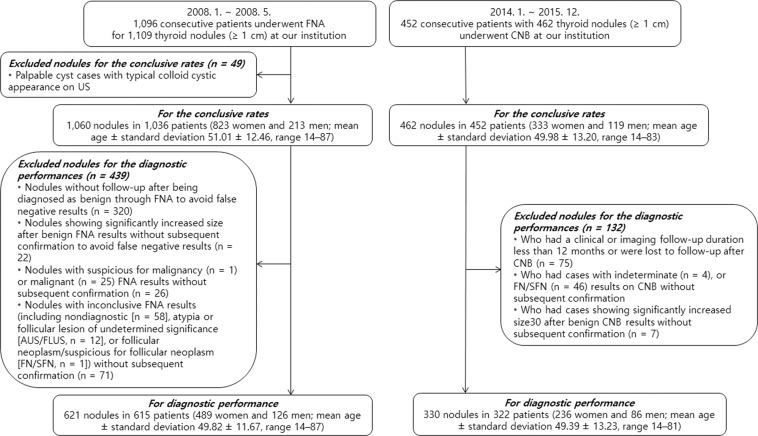
Flowchart of patient population according to inclusion and exclusion criteria. From January 2008 to May 2008, 1,096 consecutive patients underwent FNA for 1,109 thyroid nodules (≥1 cm) at our institution. The study included 1,060 nodules in 1,036 patients for the conclusive rates, and 621 nodules in 615 patients for diagnostic performance. A total of 452 consecutive patients with 462 thyroid nodules (≥1 cm) underwent CNB at our institution between January 2014 and December 2015. The study included 462 nodules in 452 patients for the conclusive rates, and 330 nodules in 322 patients for the diagnostic performance.

From January 2008 to May 2008, 1,096 consecutive patients underwent FNA for 1,109 thyroid nodules (≥1 cm) at our institution (Fig. 1). First, for the evaluation of conclusive rates, we excluded 49 palpable cyst cases with typical colloid cystic appearance on US to avoid false negative results. Second, for the assessment of diagnostic performance, we excluded 439 nodules additionally as follows: (1) 320 nodules with no follow-up after being diagnosed as benign by FNA in order to avoid false negative cases, (2) 22 nodules showing significantly increased size after benign FNA results without subsequent confirmation to avoid false negative cases, (3) 26 nodules with suspicious for malignancy (*n* = 1) or malignant (*n* = 25) FNA results without subsequent confirmation, and (4) 71 nodules with inconclusive FNA results (including nondiagnostic [*n* = 58], AUS/FLUS [*n* = 12], or FN/SFN [*n* = 1]) without subsequent confirmation^27^. Finally, the study enrolled 1,060 nodules in 1,036 patients (823 women and 213 men; mean age ± standard deviation 51.01 ± 12.46, range 14–87) for the conclusive rates, and 621 nodules in 615 patients (489 women and 126 men; mean age ± standard deviation 49.82 ± 11.67, range 14–87) for diagnostic performance.

A total of 452 consecutive patients with 462 thyroid nodules (≥1 cm) underwent CNB at our institution between January 2014 and December 2015 (Fig. 1). First, we included all 452 patients for the evaluation of conclusive rates. Second, for the assessment of diagnostic performance, we excluded 132 patients who had an imaging or clinical follow-up duration less than one year or were lost to follow-up after CNB (*n* = 75), who had cases with indeterminate (*n* = 4), or FN/SFN (*n* = 46) results on CNB without subsequent confirmation, and who had cases showing significantly increased size^27^ after benign CNB results without subsequent confirmation (*n* = 7). Finally, the study enrolled 462 nodules in 452 patients (333 women and 119 men; mean age ± standard deviation 49.98 ± 13.20, range 14–83) for the conclusive rates, and 330 nodules in 322 patients (236 women and 86 men; mean age ± standard deviation 49.39 ± 13.23, range 14–81) for the diagnostic performance.

For the assessment of diagnostic performance, final diagnoses of malignant nodules (*n* = 252) were decided based on the pathological results by surgery (*n* = 244) or CNB (*n* = 8). Final diagnoses of benign nodules (*n* = 699) were decided based on the pathological results by surgery (*n* = 196, 28.0%), benign FNA or CNB results repeated at least twice (*n* = 317, 45.4%) and a concordant benign result of FNA or CNB and a decreased or stable nodule size at US or clinical follow-up of at least one year (*n* = 186, 26.6%).

### US examination and US-Guided biopsy procedures

All US examinations were operated with a 5- to 12-MHz linear-array transducer and a real-time US system (HDI 5000, Philips Medical Systems, Bothell, WA, USA; iU22, Philips Medical Systems, Bothell, WA, USA). The US examinations and US-guided biopsy procedures were performed by four faculty radiologists who specialize in thyroid imaging (more than seven years of experience), fellows, or residents. Faculty radiologists supervised all procedures of the fellows or residents.

US-guided FNA was operated with a 23-gauge needle attached to a 2 mL disposable plastic syringe routinely and a 21-gauge needle attached to a 2 mL or 5 mL disposable plastic syringe selectively. US-CNB was performed with a disposable 18-gauge, double-action spring-activated needle with an 11 mm excursion (TSK Ace-cut; Create Medic, Yokohama, Japan) after local anesthesia. After biopsy, every patient was observed with local compression of the biopsy site for 10 to 20 minutes.

In our institution, we have used a CNB technique to contain the nodule, nodular margin, and surrounding parenchyma in at least one core specimen since June 2013 with a minimum of two cores^14,30^. Among the CNB patients, there was no case of technical failure or complication in obtaining biopsy specimens during the study period.

### Image analysis

US images were retrospectively reviewed and assessed with consensus by two faculty radiologists (S.Y.H. and J.H.S.) who didn’t know previous FNA or CNB result or final diagnosis.

US features were retrospectively classified according to the ACR TI-RADS and K-TIRADS^3,7^. All thyroid nodules were recorded for composition, echogenicity, shape, orientation, margin, and echogenic dot (calcification) according to the ACR TI-RADS or K-TIRADS. In the ACR TI-RADS, US findings are given 0–3 points corresponding to their association with malignancy, and points were added to assess the final risk stratification level. In the K-TIRADS, malignancy risk stratification was finally assessed into five categories based on the US characteristics of the thyroid nodules. The nodule size was determined as the maximum diameter measured on the static images, regardless of the acquisition plane.

### Cytology and histology analysis

During the study period, one of seven experienced pathologists reassessed the results of FNA and CNB according to the Bethesda System for Reporting Thyroid Cytopathology and the research of the Korean Endocrine Pathology Thyroid Core Needle Biopsy Study Group, respectively^15,16^.

### Statistical analysis

Significant differences were found in the characteristics and numbers between CNB and FNA nodules. Therefore, propensity score matching was conducted to match FNA patients to CNB patients on sex and age, previous inconclusive results (i.e., I [non-diagnostic] or III [AUS/FLUS or indeterminate]) of FNA or CNB, present FNA or CNB results, nodule size, nodule composition on US, ACR TI-RADS risk category, and K-TIRADS category^31^. Clinicopathological and US features between the two biopsy groups were compared with the independent two-sample *t* test (before matching) and the paired *t* test (after matching) for continuous variables, and the χ^2^ test or Fisher’s exact test (before matching) and McNemar’s test (after matching) for categorical variables.

The sensitivities, specificities, PPVs, negative predictive values (NPV), and accuracies of FNA and CNB were calculated for the diagnosis of thyroid malignancy (i.e. Bethesda or CNB diagnostic category V and VI vs. II) and for the determination of surgical indication (i.e. Bethesda or CNB diagnostic category IV, V, and VI vs. I, II, and III)^32^, and we compared them using the χ^2^ test or Fisher’s exact test in the non-matched population and generalized estimating equations in the matched population.

Conclusive rates for FNA and CNB (i.e. Bethesda or CNB diagnostic category II, IV, V, and VI vs. I and III) were also investigated and compared according to the final US category and nodule size using the χ^2^ test or Fisher’s exact test in the non-matched population and McNemar’s test for categorical variables in the matched population^32^.

Statistical significance was accepted with a two-tailed P value < 0.05. All statistical analyses were conducted using SAS version 9.4 (SAS Institute, Cary, NC, USA) and R 3.2.2 (Vienna, Austria; http://www.R-project.org/).
